# Small-Molecule Antibiotics Inhibiting tRNA-Regulated Gene Expression Is a Viable Strategy for Targeting Gram-Positive Bacteria

**DOI:** 10.1128/AAC.01247-20

**Published:** 2020-12-16

**Authors:** Ville Y. P. Väre, Ryan F. Schneider, Haein Kim, Erica Lasek-Nesselquist, Kathleen A. McDonough, Paul F. Agris

**Affiliations:** a Department of Biological Sciences, University at Albany-SUNY, Albany, New York, USA; b Department of Biomedical Sciences, School of Public Health, University at Albany-SUNY, Albany, New York, USA; c Department of Medicine, Duke University School of Medicine, Durham, North Carolina, USA; d Wadsworth Center, New York State Department of Health, Albany, New York, USA

**Keywords:** mRNA target for antibiotics, tRNA gene regulation, Gram-positive pathogens, novel small-molecule antibiotics, antibiotic resistance, synergy with common antibiotics

## Abstract

Bacterial infections and the rise of antibiotic resistance, especially multidrug resistance, have generated a clear need for discovery of novel therapeutics. We demonstrated that a small-molecule drug, PKZ18, targets the T-box mechanism and inhibits bacterial growth. The T-box is a structurally conserved riboswitch-like gene regulator in the 5′ untranslated region (UTR) of numerous essential genes of Gram-positive bacteria. T-boxes are stabilized by cognate, unacylated tRNA ligands, allowing the formation of an antiterminator hairpin in the mRNA that enables transcription of the gene. In the absence of an unacylated cognate tRNA, transcription is halted due to the formation of a thermodynamically more stable terminator hairpin. PKZ18 targets the site of the codon-anticodon interaction of the conserved stem I and reduces T-box-controlled gene expression. Here, we show that novel analogs of PKZ18 have improved MICs, bactericidal effects against methicillin-resistant Staphylococcus aureus (MRSA), and increased efficacy in nutrient-limiting conditions. The analogs have reduced cytotoxicity against eukaryotic cells compared to PKZ18. The PKZ18 analogs acted synergistically with aminoglycosides to significantly enhance the efficacy of the analogs and aminoglycosides, further increasing their therapeutic windows. RNA sequencing showed that the analog PKZ18-22 affects expression of 8 of 12 T-box controlled genes in a statistically significant manner, but not other 5′-UTR regulated genes in MRSA. Very low levels of resistance further support the existence of multiple T-box targets for PKZ18 analogs in the cell. Together, the multiple targets, low resistance, and synergy make PKZ18 analogs promising drugs for development and future clinical applications.

## Abstract (precis)

Bacterial infections and the rise of antibiotic resistance, especially multidrug resistance, have generated a clear need for discovery of novel therapeutics. We demonstrated that a small-molecule drug, PKZ18, targets the T-box mechanism and inhibits bacterial growth. The T-box is a structurally conserved riboswitch-like gene regulator in the 5′ untranslated region (UTR) of numerous essential genes of Gram-positive bacteria. T-boxes are stabilized by cognate, unacylated tRNA ligands, allowing the formation of an antiterminator hairpin in the mRNA that enables transcription of the gene.

## INTRODUCTION

The World Health Organization (WHO) and the Centers for Disease Control and Prevention (CDC) consider antibiotic resistance one of the greatest global public health challenges (1; https://www.who.int/en/news-room/fact-sheets/detail/antibiotic-resistance). The spread of hospital-acquired infections, including Clostridioides difficile and methicillin-resistant Staphylococcus aureus (MRSA), is a major concern for public health. There is ongoing research and debate on therapeutic treatments and prevention of bacterial, especially nosocomial, infections (1, 2). However, the WHO estimates that current efforts for curbing antimicrobial resistance are insufficient to overcome the problem (https://www.who.int/en/news-room/fact-sheets/detail/antibiotic-resistance), so research and development of unique targets and novel antibiotics are crucial.

Mainstays of antibiotic therapy against bacterial pathogens involve blocking bacterial growth, either by inhibiting cell wall, protein, or DNA syntheses, or by hindering critical metabolic processes. The majority of current antibiotics affect the same cellular processes that have been targeted by previous iterations of the various classes of antibiotics (3). Unfortunately, drug effectiveness has been severely compromised due to rapid emergence of resistance. In the case of S. aureus, resistance readily occurs through a variety of mechanisms, such as enzymatic inactivation, altered binding affinities, antibiotic trapping, efflux pumps, acquisition of chromosomal cassettes (*mec* elements), or spontaneous mutation with positive selection, in response to the exposure of each new antibiotic (4, 5). Four new antibiotics were approved by the U.S. Food and Drug Administration (FDA) in 2018, but all are new iterations of antibiotics from existing classes; hence, emergence of resistance is likely (6). Strategies to increase the effectiveness of our current antibiotic repertoire include several recent studies reporting synergistic effects of antibiotics, which can increase the life span of the drugs before resistance emerges (7–9). The use of natural products in combination with small-molecule antibiotics may provide an additional avenue for targeting resistant bacteria (10). As combinatorial usage of antibiotics becomes more common (7, 9), industrial interest in novel synthesis of drug combinations that work synergistically increases (11). At the same time, discovering new drug targets is paramount. One promising target for drug discovery is that of riboswitches, with several drug discovery platforms already being established for targeting them (12, 13).

Individual riboswitches, RNA elements that control transcription or translation, are specific domains within mRNAs that respond to metabolites or ions. Natural small-molecule metabolites bind specifically and tightly to riboswitches in 5′ untranslated regions (5′ UTRs) of messenger RNAs (mRNA), altering conformation and regulating transcription elongation or translation initiation. Thus, analogs of a metabolite can have a lethal effect on the expression of a single essential gene (14–17). Resistance to antibiotics that target a single riboswitch can occur frequently, and strains resistant to an inhibitor of the riboflavin riboswitch have readily emerged (18–21). However, inhibitors of bacterial aminoacyl-tRNA synthetases (aaRS) have been proven effective (22), as have small-molecule inhibitors of RNA functions (15). The T-box, a conserved riboswitch-like RNA element, presents a target that combines these two options for antibacterial intervention.

**FIG 1:**
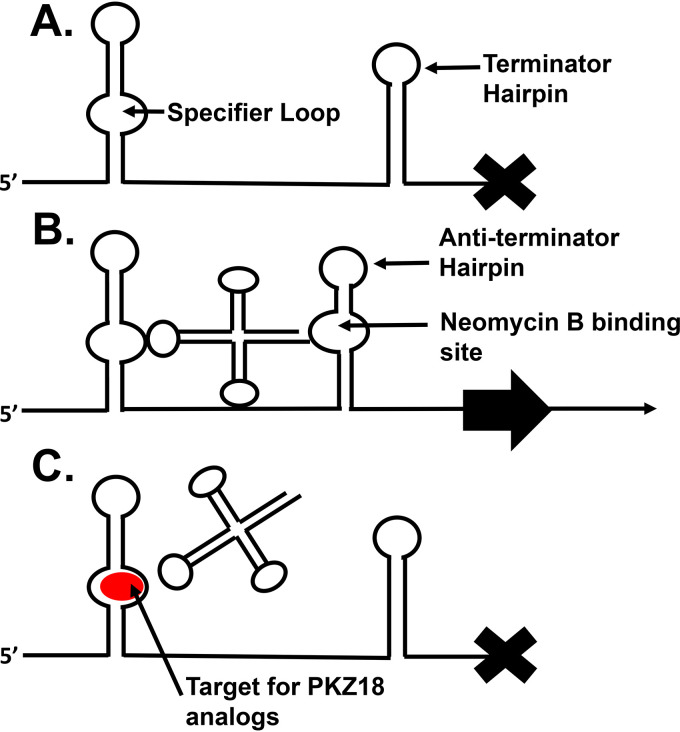
Simplified T-box model. Additional T-box hairpins and apical loop to tRNA elbow interaction are not shown. (A) An aminoacylated tRNA cannot stabilize the antiterminator helix, and the thermodynamically more stable terminator hairpin is formed, causing termination of transcription. (B) A cognate, unacylated tRNA can stabilize the antiterminator helix, which allows transcription to continue. (C) PKZ analogs binding to the specifier loop prevent codon-anticodon interaction resulting in transcriptional termination.

First discovered in 1992 (23), T-boxes are found in the 5′ UTRs of multiple genes or operons of Gram-positive, but not Gram-negative, bacteria. The T-box is a conserved and dynamic RNA structure that recognizes a cognate tRNA as its ligand through a codon-anticodon interaction where the codon is found in the specifier loop of stem I of the T-box (Fig. 1) (24–26). T-boxes control the transcription of multiple essential genes in common pathogens such as S. aureus and C. difficile (24, 25). In addition, a novel class of translation-regulating T-boxes in *Actinobacteria* utilizes a tRNA to stabilize a helix complementary to the Shine-Dalgarno (SD) sequence that allows the ribosomal binding site or Shine-Dalgarno sequence to be exposed (26, 27). The solution, crystal, and cryo-electron microscopy (cryo-EM) structures have been determined for multiple different T-boxes from several organisms and show that a structure containing the specifier loop in the first mRNA hairpin, stem I, is conserved (28–31). Specific tRNAs initially bind to a cognate codon in the specifier loop. An unacylated cognate tRNA can then stabilize an antiterminator helix in the mRNA downstream of stem I through base-pairing interactions with the conserved 3′ NCCA acceptor end of the tRNA and a conserved complementary sequence 5′-UGGN of the mRNA, the “T-box,” thus, allowing the RNA polymerase to continue transcription (Fig. 1B). Conversely, charged or even slightly modified tRNA cannot stabilize the antiterminator helix, causing the thermodynamically more stable terminator hairpin to form and transcription to halt (Fig. 1) (24–26, 32).

Drugs targeting tRNA-dependent gene regulation have been investigated by us and others. Hines and colleagues showed that the antiterminator “T-box” can be targeted with substituted oxazolidinones (12, 33–38). We previously demonstrated that PKZ18 selectively targets stem I specifier loops in Gram-positive bacteria and directly reduces T-box transcriptional read-through of the associated genes. PKZ18 prevents the codon-anticodon reading required for tRNA binding and is refractory to resistance. However, the MIC of PKZ18 (32 to 64 μg/ml) against most Gram-positive bacteria is higher than desirable, and the drug shows cytotoxicity against eukaryotic cells at the MIC after 48 hours. Furthermore, PKZ18 is only bacteriostatic against clinical isolates of methicillin-resistant Staphylococcus aureus (MRSA) (33). While PKZ18 provides an important proof of principle showing that small-molecule antibiotics are suitable for targeting the T-box mechanism, further development is required. In this study, we characterize PKZ18 analogs with improved targeting of the T-box mechanism and reduced cytotoxicity. Use of these PKZ18 analogs in combination with aminoglycoside antibiotics increased the efficacy of both drugs by 4- to 8-fold. Also, we demonstrate that one analog, PKZ18-22, inhibits the expression of 8 of the 12 T-boxes in MRSA.

## RESULTS

### PKZ18 binds specifically to the specifier loop.

We previously reported that PKZ18 binds with low micromolar dissociation constants to chemically synthesized truncated stem I constructs of the Bacillus subtilis *glyQS* and *tyrS* T-boxes (33). However, the mass spectrometric analysis did not determine whether the PKZ18 had bound the specifier loop or the stem I in a generic RNA interaction. Analysis of the UV thermodynamics of two new stem I constructs (Fig. S1A and B in the supplemental material) confirmed binding to the stem I specifier loop. PKZ18 thermally destabilized the RNA (change in melting temperature [Δ*T_m_*], −18.52 °C; free energy [ΔΔ*G*], +3.41 kcal mol^−1^) when bound to the RNA comprised of the wild-type sequence of the truncated *glyQS* stem I with the specifier loop. However, PKZ18 did not affect the thermodynamic parameters of a similarly sized construct that has the *gly* codon but lacks the specifier loop (Δ*T_m_*, −0.33; ΔΔ*G*, +0.22) (Fig. S1C). This result establishes that PKZ18 binding to stem I occurs within the specifier loop.

### New analogs have improved activity and bactericidal effects.

Small changes in the chemical structure of a putative drug can influence activity. We compared PKZ18 analogs with minor variations in chemical structure to better define moieties that contribute to increased efficacy (Table 1). Here, we found that PKZ18-22 is bactericidal against both B. subtilis 168 and MRSA (Table 1) and this activity correlates with a reduced MIC due to the extension of the carbon tail on the benzene *para* to the thiazole. The reduced MIC, however, was lost by changing the bridge C7 of the norbornane to an oxygen. Comparison of PKZ18-54 with PKZ18-57 suggests that a straight aliphatic chain *para* to the thiazole enhances activity (PKZ18-54) compared to that of a branched chain (PKZ18-57). Increasing the polarity, as with the ether in PKZ18-55, nullified activity. The substitution of norbornene for the norbornane maintained the bioactivity, making further derivatizations possible by way of additions to the double bond. The methyl group at position 5 of the thiazole, or the absence thereof, had no effect on activity, as shown by MIC and minimal bactericidal concentration (MBC) values with PKZ18-22 and PKZ18-53, confirming previous observations (33).

**TABLE 1:**
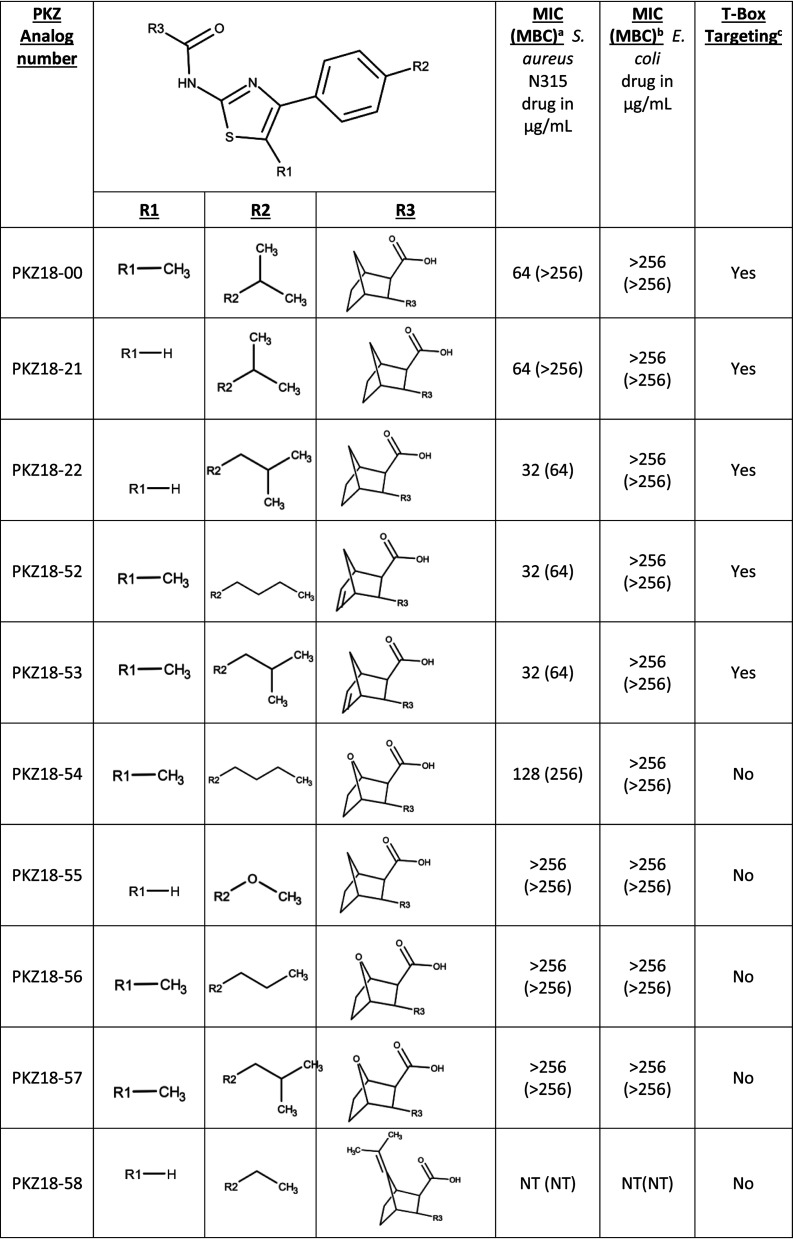
Structures of PKZ18 analogs^*d*^

a Drug concentrations are represented in micrograms per milliliter against S. aureus N315 and E. coli.

b Gram-negative control-showing drugs do not target an organism that does not contain T-boxes.

c Confirmation of inhibition of T-box mechanism from qRT-PCR assay.

d Different chemical moieties are listed corresponding to the three locations where changes were made. The MIC and MBC against the Gram-positive MRSA, as well as the Gram-negative E. coli, are shown. PKZ18-52 and PKZ18-53 have a norbornene moiety instead of norbornane (R3).

We previously observed that PKZ18 is active against B. subtilis 1A5 grown in minimal media at much lower concentrations than the reported MIC (33). In contrast, media-dependent antimicrobial activity against Escherichia coli has been reported by others where the antibiotic loses efficacy in nutrient-limiting conditions (39). To measure the effect of growth media on activity, we compared the MIC of parent PKZ18 against both B. subtilis 168 and S. aureus 4220 in rich versus minimal media. The MICs of PKZ18 were 8- (B. subtilis 168) and 4-fold (S. aureus 4220) lower when the cultures were grown in minimal versus rich media. Similarly, PKZ18-22 exhibited a 4-fold reduction in MIC, while PKZ18-52 and PKZ18-53 had 2-fold reductions against the MRSA strain S. aureus N315 in minimal compared to rich media (Table 2). In contrast, gentamicin and mupirocin had increased MICs in minimal media compared to rich media against S. aureus N315 (Table 2).

**TABLE 2:** Media-dependent activity of drugs^*d*^

Drug	MIC (μg/ml) against B. subtilis 168 in:		MIC (μg/ml) against S. aureus in:	
	Rich media	Minimal media	Rich media	Minimal media
PKZ18	64	8	64^*a*^	16^*a*^
PKZ18-22	16	4	32^*b*^	8^*b*^
PKZ18-52	NT^*c*^	NT	16–32^*b*^	16^*b*^
PKZ18-53	NT	NT	32^*b*^	16^*b*^
Gentamicin	0.125	0.25	0.5^*b*^	2.0^*b*^
Mupirocin	NT	NT	0.25^*b*^	1.0^*b*^

a S. aureus 4220.

b S. aureus N315.

c NT, not tested.

d PKZ18 analogs are more effective in minimal media against both B. subtilis and S. aureus, whereas gentamicin and mupirocin are more effective in rich media.

### Broad T-box targeting by PKZ18 analogs.

**FIG 2:**
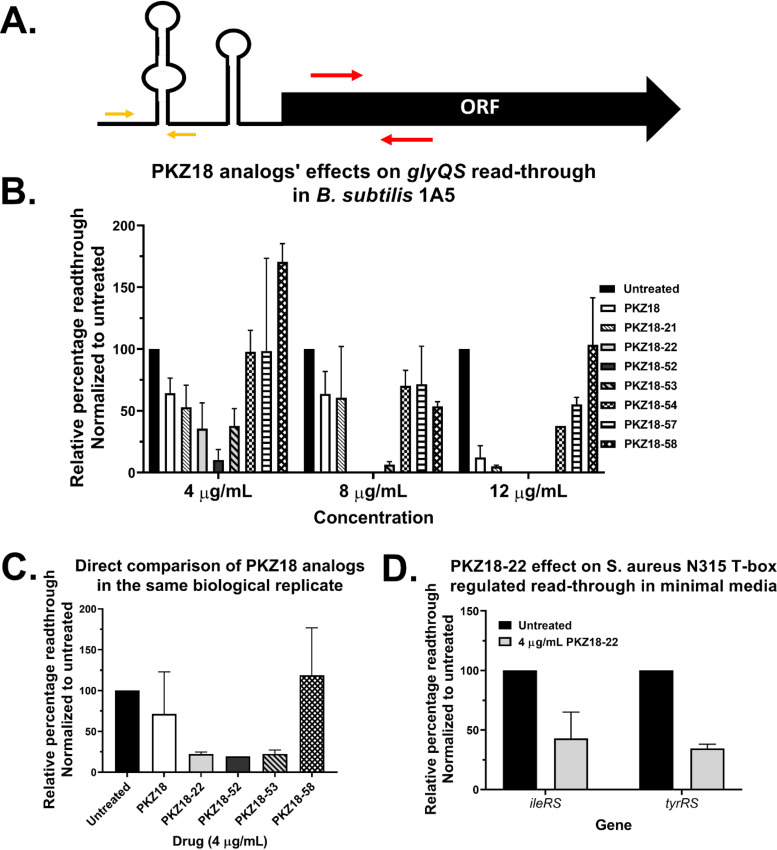
PKZ18 analogs’ effect on transcriptional read-through in minimal media. (A) Sketch of a T-box-regulated gene layout where the two primers used for testing are represented by arrows for the T-box containing 5’UTR (yellow) and the ORF (red). (B) Effect of PKZ18 analogs on transcriptional read-through on B. subtilis *glyQS*. Concentration is shown on the *x* axis, and an absence of a bar indicates no observed growth at that concentration. (C) Side-by-side treatment of B. subtilis 1A5 with different PKZ18 analogs on the same biological replicate showing a direct comparison of the novel analogs’ improved activity compared to PKZ18 as measured by reduced read-through of *glyQS*. Due to a limited amount of PKZ18-52, the bar is representative of one biological replicate. (D) Effect of PKZ18-22 on transcriptional read-through on the T-box-regulated *ileS* (*n* = 3) and *tyrS* (*n* = 2) in WT MRSA.

PKZ18-21, PKZ18-22, PKZ18-52, and PKZ18-53 inhibited T-box-controlled expression of *glyQS* in the glycine auxotroph B. subtilis 1A5, with increasing concentrations showing increased termination of read-through by reverse transcription-quantitative PCR (qRT-PCR) (Fig. 2A and B). Additionally, PKZ18-22, PKZ18-52, and PKZ18-53 inhibited B. subtilis 1A5 read-through significantly better than PKZ18 (at 4 μg/ml of each drug) when the bacteria were grown side by side in the same biological replicate assay (Fig. 2C). These novel analogs inhibited culture growth, and no RNA could be obtained at 12 μg/ml where PKZ18 was previously shown to be most active (33).

**FIG 3:**
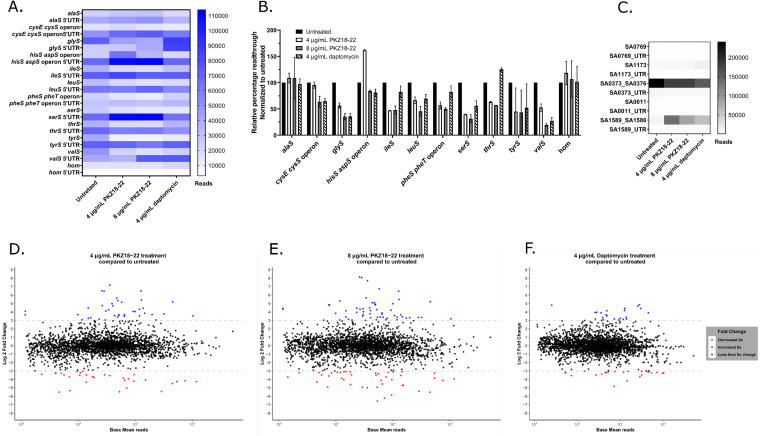
RNA sequencing of MRSA showing the mean of two biological replicates grown in minimal media. (A) A heatmap of the expression of the 12 T-box controlled genes or operons in S. aureus and their respective 5’UTRs (T-boxes) showing a comparison of the three treatments’ effect on initiation (5’UTR) and read-through (ORF). (B) Data from panel A showing relative read-through (normalized to untreated and presented as a column graph). (C) Heatmap of some other 5’UTR regulated genes: SA0769 and SA0011 are regulated by SAM riboswitches, SA1173 is a sigma B regulated CDS, SA0373 is regulated by a purine riboswitch, and SA1589 is a *trans*-encoded sRNA. (D to F) Comparison of antibiotic effect on overall change in gene expression in MRSA is shown: (D) 4 μg/ml PKZ18-22 to untreated, (E) 8 μg/ml PKZ18-22 to untreated, and (F) 4 μg/ml daptomycin to untreated.

We used RNA sequencing to determine if native expression of T-box-controlled genes was affected by PKZ18-22 in MRSA. We compared the expression of the 5′ UTR of T-box-regulated genes to the expression of their open reading frames (ORFs). The expression of 8 out of the 12 genes under T-box control was reduced, one remained at untreated levels when tested at 8 μg/ml PKZ18-22, and three remained at untreated levels with either 4 or 8 μg/ml PKZ18-22 (Fig. 3A), indicating an inhibitory effect by PKZ18-22 on the T-box-regulated genes with decreased expression. Elevated 5′-UTR expression for several T-box-mediated genes following PKZ18-22 treatment further indicated an increase in transcriptional initiation with early termination in the presence of PKZ18-22 (Fig. 3A). We then ratioed the reads from the ORF to the read numbers in the 5′ UTR for each gene to represent relative read-through and normalized the data to the untreated control. There was a reduction in read-throughs of *glyS* (*P* = 0.004311), i*leS* (*P* = 0.010565), *leuS* (*P* = 0.013825), *pheST* (*P* = 0.001594), *serS* (*P* = 0.005975), *thrS* (*P* = 0.000019), and *valS* (*P* = 0.000566), indicating inhibition of these T-boxes by both concentrations of PKZ18-22; the *P* values shown in parentheses after each gene are for 8 μg/ml treatment based on a *t* test comparing the read-through to the untreated sample (Fig. 3B). These were also all significant hits for the 8-μg/ml PKZ18-22 treatment (Fig. S2). Expression and read-through of *tyrS* was reduced, but the read-through reduction was not statistically significant (Fig. 3A and B). The cysteine and histidine T-boxes (*cysE-cysS* and *hisS-aspS* operons, respectively) were inhibited only by the higher concentration of PKZ18-22. Read-through of *alaS* was not inhibited, but 8 μg/ml of PKZ18-22 was sufficient to maintain expression at the same level as 4 μg/ml in spite of increased transcription initiation (Fig. 3A and B). The only T-box-regulated gene in MRSA that does not transcribe an aaRS gene, *hom*, is preceded by a methionine T-box (24) and was expressed at counts too low to allow conclusions. We used 4 μg/ml of daptomycin as a control antibiotic. It did not significantly affect initiation of T-box-controlled genes, but expression and read-through with daptomycin were lower than untreated for many of the genes (Fig. 3A and B).

At least 40 genes or operons are controlled by riboswitches or riboswitch-like RNA elements in S. aureus (40). We confirmed the T-box-specific activity of PKZ18-22 by comparing the 5′ UTR expression and ORF expression of other riboswitch-controlled genes. No clear trends with PKZ18-22 treatment were evident, and relative read-through of the tested genes did not significantly change with treatment (Fig. 3C). Treatment was expected to cause a large depletion of aminoacylated tRNAs with the possibility of pleiotropic changes in native gene expression as a stress reaction. Treatment with PKZ18-22 resulted in a large overall change in expression, which was similar to that associated with daptomycin (Fig. 3D to F). Volcano plots of the three treatments also indicate a large pleiotropic effect by all treatments (Fig. S2A to C).

We confirmed the RNA sequencing results of PKZ18-22 with qRT-PCR. Comparison of the relative read-through of *ileS* and *tyrS* in MRSA grown in minimal media showed that PKZ18-22 at 4 μg/ml caused a decrease in the read-through of both genes, consistent with PKZ18-22 working as an inhibitor of the T-box mechanism (Fig. 2D). A similar but less pronounced trend was observed when MRSA was grown in rich media and treated with 8 μg/ml of PKZ18-22. The read-through of both *ileS* and *tyrS* was reduced compared to that in untreated MRSA (Fig. S3). However, expression of the genes is reduced in nutrient-rich conditions, as the pool of uncharged tRNAs tends to be relatively low as well.

### Synergistic activity of PKZ18 analogs with different antibiotics.

Combinatorial usage of antibiotics is widespread in clinical settings, and aminoglycosides are known to enhance the activity of drugs that deplete the pool of charged tRNAs (7–9, 41), so we tested the possibility of increased efficacy resulting from synergistic interactions between several clinically relevant antibiotics and PKZ18 analogs. PKZ18-22 was synergistic with neomycin and kanamycin (fractional inhibitory concentration [FIC], 0.38), and PKZ18-22, PKZ18-52, and PKZ18-53 were all synergistic with gentamicin (FIC, 0.38) (Table 3). However, interaction between streptomycin and PKZ18-22 was only additive (FIC, 0.75). Additionally, both the beta-lactam antibiotic ampicillin and the ribosome-targeting chloramphenicol showed additive effects with PKZ18-22 and PKZ18-53 (FIC, 0.75). No combinatorial effects were observed with PKZ18 analogs and the other drugs tested, including some antibiotics commonly used to treat MRSA, namely, mupirocin, vancomycin, oxacillin, and daptomycin. No antagonistic interactions with PKZ18 analogs were discovered.

**TABLE 3:** Combinatorial effects of PKZ18 analogs with various antibiotics^*a*^

Antibiotic class	Drug tested	Primary target	MIC alone (μg/ml)	MIC with PKZ18 analog (μg/ml)	PKZ18 analog used	Amt used (μg/ml)	Fold change	FIC	Effect
Aminoglycosides	Gentamicin	16S rRNA (A-site) codon-anticodon reading	0.50–1.00	0.031	18-22, 18-52, 18-53	8.000	8	0.38	Synergistic
	Streptomycin	16S rRNA (A-site) and S12 initial tRNA selection	4.000	2.000	18-22	8.000	2	0.75	Additive
	Hygromycin	16S rRNA mRNA translocation	64.000	32.000	18-22	8.000	2	0.75	Additive
	Kanamycin	16S rRNA and S12 wobble base pairing	256.000	64.000	18-22	4.000	4	0.38	Synergistic
	Neomycin	16S rRNA (A-site) codon-anticodon reading	256.000	32.000	18-22	8.000	8	0.38	Synergistic
Beta-lactams	Ampicillin	Transpeptidase (cell wall)	16-32	8.000 4.000	18-22, 18-53	16.000	4	0.75	Additive
	Oxacillin	Transpeptidase (cell wall)	0.500	0.500	18-22	NA	1	1.00	Indifferent
Glycopeptides	Vancomycin	NAM/NAG-peptides (cell wall)	0.500	0.500	18-22	NA	1	1.00	Indifferent
Quinolones and fluoroquinolones	Ofloxacin	Topoisomerases and gyrase	0.250	0.250	18-22	NA	1	1.00	Indifferent
Tetracyclines	Tetracycline	Ribosomal A-site	0.250	0.250	18-22	NA	1	1.00	Indifferent
Other	Rifampin	RNA polymerase	0.025	0.025	18-22	NA	1	1.00	Indifferent
	Daptomycin	Membrane permeability (cell wall)	4.000	4.000	18-22	NA	1	1.00	Indifferent
	Mupirocin	ileRS	0.125	0.125	18-22	NA	1	1.00	Indifferent
	Chloramphenicol	50S subunit (23S rRNA)	64–128	32.000	18-22, 18-53	8.000	2	0.75	Additive

a Class-specific antibiotics, and their respective targets are listed. The MIC and MIC with PKZ18 analog are shown, and where multiple analogs were tested, they are shown in the same cell. Fold change refers to the standalone MIC to combinatorial MIC ratio of the antibiotics tested in conjunction with PKZ18 analogs. FIC refers to the fiduciary inhibitory concentration, a measurement of synergy, and the value is explained in the effect column.

The synergy observed between PKZ18-22 and gentamicin was substantial, with a 4-fold decrease in the MIC of PKZ18-22 and an 8-fold decrease in the MIC of gentamicin. Similar effects were observed for the combinations of PKZ18-22 with neomycin or with kanamycin, PKZ18-52 with gentamicin, and PKZ18-53 with gentamicin. Due to limited supply of the novel analogs, not all combinations were tested with all the analogs; however, the analogs used replicated the effect PKZ18-22 had on the MICs of the drugs with which they were tested.

### Cytotoxicity.

**FIG 4:**
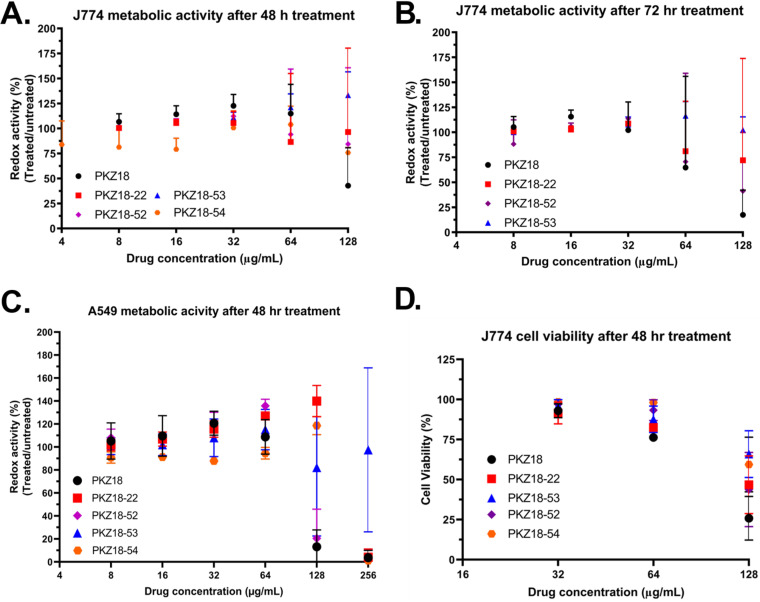
Cytotoxicity of PKZ18 analogs. All values are normalized to an untreated sample. A drug concentration of 0 corresponds to the DMSO control used in each experiment. (A) J774.16 murine macrophage metabolic activity as measured by alamarBlue after 48 hours of treatment. (B) J774.16 murine macrophage metabolic activity as measured by alamarBlue after 72 hours of treatment. (C) A549 human lung epithelial cells’ metabolic activity as measured by alamarBlue after 48 hours of treatment. (D) J774.16 murine macrophage viability measured by Trypan blue cell counting after 48 hours of treatment.

The parent compound PKZ18 is moderately cytotoxic at MIC level (33), so finding analogs with both improved efficacy and therapeutic window of cytotoxicity was of paramount interest to us. We tested our small set of novel analogs against A549 human lung epithelial cells as well as against J774.16 murine macrophages by measuring both the redox potential of alamarBlue as an indicator of metabolic function and trypan blue staining for cell viability. After 48 hours of treatment, PKZ18 increased cellular redox activity at 64 μg/ml but decreased after 72 hours (Fig. 4A and B), and PKZ18 caused a near-total reduction of redox activity at 128 μg/ml after 48 hours (Fig. 4A and C), i.e., cytotoxicity at 2-fold above the MIC, agreeing with our previously reported cytotoxicity data (33). Other analogs showed increased redox activity at 128 μg/ml and a complete loss of redox activity at 256 μg/ml, indicative of stress and killing, respectively (Fig. 4C). The data are consistent between the two cell lines tested, although we were not able to test J774.16 cells at 256 μg/ml due to their low tolerance to dimethyl sulfoxide (DMSO) (Fig. 4A and B). The data from the alamarBlue assay indicated lower cytotoxicity of the analogs than the parent compound PKZ18, where PKZ18 showed cytotoxicity at the MIC, and the analogs were 2- to 4-fold above the MIC.

Cell viability measured by trypan blue cell staining of the macrophages showed that the cells retained a 76% viability with PKZ18 at 64 μg/ml, while there was 82 to 98% cell viability with PKZ18-22, PKZ18-52, PKZ18-53, and PKZ18-54. Macrophage viability was only 25% after treating with 128 μg/ml PKZ18 for 48 hours, but it was 43 to 65% with the other analogs (Fig. 4D).

The synergistic concentrations at which the PKZ18 analogs were effective with the aminoglycosides, as well as ampicillin and chloramphenicol, showed no change in metabolic activity of A549 cells, indicating that the combinatorial concentrations required for inhibiting bacterial growth are nontoxic to human lung epithelial cells (Fig. S4).

### Mutation frequency of PKZ18-22 and characterization of resistant mutant.

We previously reported that resistance is less likely to emerge against PKZ18 and its analog PKZ18-22 than presently used antibiotics because the PKZ18 family of compounds targets multiple T-box-regulated genes (33). To measure resistance in a more clinically relevant setting, we tested PKZ18-22 or gentamicin against MRSA and used B. subtilis as a control. MRSA exposed to MBC levels (64 μg/ml) of PKZ18-22 did not readily form resistant colonies, with only one colony recovered from a total of 1.8 × 10^11^ CFU plated, giving a resistance frequency of 5.6 × 10^−12^. In comparison, MRSA plated on 10 μg/ml gentamicin (20× MIC and 5× MBC against MRSA) showed a resistance frequency of 1.6 × 10^−7^ (Table 4). This clearly demonstrates that PKZ18-22 is highly refractory to resistance, consistent with the RNA sequencing results that PKZ18-22 targets multiple T-boxes simultaneously. No PKZ18-resistant colonies were found. Additionally, B. subtilis did not generate resistant colonies against either PKZ18 or PKZ18-22. The mutant emerging from the fluctuation assay (named PKZRSA1) was sequenced for the tyrosine T-box, but no mutations were found. We confirmed the mutant was resistant with a kill curve assay where the mutant, but not the wild type (WT), grew through in the presence of PKZ18-22 (Fig. S5A). The levels of live cells compared to optical density (OD) remained constant with 64 μg/ml of PKZ18-22 and slightly decreased with 128 μg/ml, but both concentrations caused the number of live WT MRSA relative to optical density to decrease (Fig. S5B), indicating a truly resistant mutant. We used an ethidium bromide fluorescence-based assay to measure efflux and influx (42) to determine if PKZRSA1 had altered influx or efflux activity. No differences in either efflux or influx were observed, so this is unlikely to be the cause for resistance (Fig. S5C and D, respectively).

**TABLE 4:** Resistance frequency of PKZ18, PKZ18-22, and gentamycin against MRSA^*a*^

Drug	S. aureus N315			B. subtilis 168		
	MIC (MBC [μg/ml])	Drug concn (μg/ml)	Resistance frequency	MIC (MBC [μg/ml])	Drug concn (μg/ml)	Resistance frequency
PKZ18	64 (NA)^*b*^	128	<1.0 × 10^−11^^*a*^	64 (64)	128	<1.0 × 10^−11^
PKZ18-22	32 (64)	64	5.43 × 10^−12^	32 (64)	64	<1.0 × 10^−11^
Gentamicin	0.5 (2)	10	1.6 × 10^−7^	0.5 (2)	1	6.2 × 10^−8^

a <, no colonies were found with the number of bacteria plated.

b NA, not available.

PKZRSA1 showed a 2-fold increase in the MIC against all PKZ18 analogs tested and also exhibited a 2-fold increase in MIC against gentamicin. We screened PKZRSA1 against a wide variety of antibacterial compounds to see if it was more resistant against other drugs. The MICs of the aminoglycosides rose 2- or 4-fold against the mutant compared to the WT, and the MICs of the cell wall-targeting antibiotics vancomycin and oxacillin also increased. Vancomycin had a 2-fold increase in its MIC against the mutant, whereas 16 times more oxacillin was needed to inhibit the growth of PKZ18-22-resistant MRSA than WT MRSA. Mupirocin, tetracycline, daptomycin, and rifampin, however, showed no change in the MIC between WT and PKZRSA1. Surprisingly, the MIC of chloramphenicol was 2-fold lower for PKZRSA1 than the WT MRSA (Table 5). The media-dependent activity of antibiotics was not tested against PKZRSA1; however, the FIC of PKZ18-22 and gentamicin was 0.38 in both rich and minimal media against WT MRSA (data not shown).

**TABLE 5:**
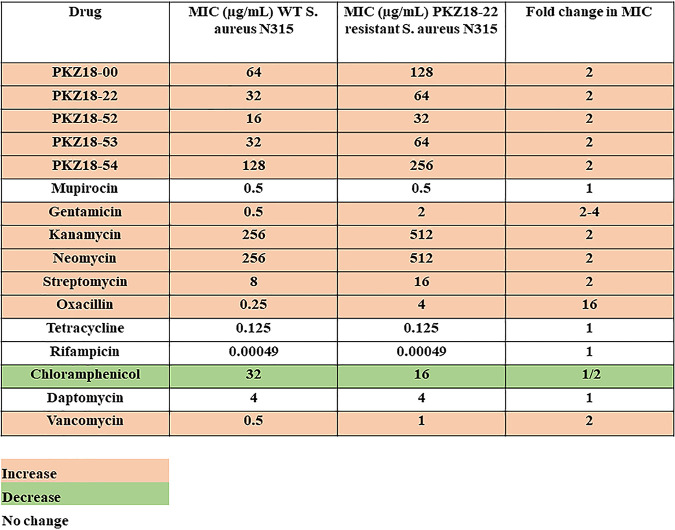
The MICs of various drugs against WT MRSA and PRSA1^*a*^

a PKZ18 analogs and aminoglycosides have increased MICs against PKZ18-22-resistant MRSA. Fold change is the MIC of PRSA1 divided by the MIC of WT MRSA. Pink indicates an increased MIC for the mutant, green represents a decrease in MIC, and white indicates no change.

## DISCUSSION

Our previous work identified PKZ18 as the parent chemotype of a promising drug. PKZ18 binding to the stem I specifier loop causes destabilization and disruption of T-box function (33, 43). However, the small therapeutic window of PKZ18 and its moderate levels of cytotoxicity have limited its applications (33). Novel PKZ18 analogs characterized in the present study address both issues by having reduced toxicity and larger therapeutic windows. Moreover, combinatorial usage of PKZ18 analogs with aminoglycosides significantly reduced the concentrations of both drugs needed to inhibit bacterial growth, further increasing the therapeutic window of these drugs. The 8-μg/ml MIC of PKZ18-22, in combination with gentamicin (approximately 20 μM), showed no cytotoxicity against eukaryotic cells and significantly lowered the effective dose of gentamicin. Aminoglycosides can have drastic side effects, including nephrotoxicity and ototoxicity that leads to permanent damage to the inner ear (44), so addition of PKZ18 analogs has the potential to enhance the therapeutic effectiveness of aminoglycosides at less toxic levels.

While these PKZ18 analogs retained some toxicity at higher concentrations, PKZ18 analogs alone or as a topical formulation with an aminoglycoside could be a potent therapy for skin infections caused by Gram-positive bacteria such as MRSA. Drugs that can be toxic systemically or do not meet the Lipinski rule of five are commonly used as topical treatments (7, 44). For example, mupirocin is used as a 2% (0.4 M) solution to treat skin infections (45), and the formulation for neomycin is 0.5% by mass (3 mM) (46). The enhanced activity of PKZ18 analogs in nutrient-limited conditions may provide an additional benefit for treatment. Antibiotics need to be functional in nutrient-limited growth environments such as the skin, where the body attempts to gain nutritional immunity and nutrient depletion for pathogens during infection (47, 48). The observed synergy of PKZ18 analogs with some aminoglycosides may be especially useful in this context, as we found that aminoglycosides were less effective in nutrient-limited environments than in rich media.

The reduction in MIC values of the novel analogs relative to the parent compound coupled with the bactericidal activity against pathogenic bacteria such as MRSA represents considerable improvement of T-box-targeting drugs over previous work. The increased effectiveness of PKZ18 analogs in minimal versus rich media is also consistent with the regulation of T-box genes. The T-box mechanism of tRNA-dependent gene expression is conserved in Gram-positive bacteria, but the number of T-boxes varies among different bacterial species and strains (24, 25). The T-box mechanism contains a codon sequence that identifies the tRNA through a codon-anticodon interaction, and the codons most sensitive to starvation in E. coli (49) are also the codons most commonly found in the specifier loops of T-boxes (43) of Gram-positive bacteria. Since the codons most sensitive to starvation are found in T-boxes, inhibition of the T-box mechanism by PKZ18 analogs may sensitize bacteria to starvation, partially explaining the enhanced effect. Additionally, there is a bias for codons ending in C in the T-box (24, 43). For example, the isoleucine T-box is the most abundantly used T-box across different Gram-positive bacterial species (24, 43), and the codon found in T-boxes is most commonly AUC (24), the most abundant and starvation-sensitive codon in E. coli (49). Conversely, aminoglycoside antibiotics exhibited higher MIC values in minimal than in rich media against both B. subtilis and S. aureus N315, indicating that higher concentrations of aminoglycosides are required to inhibit bacterial growth in nutrient-limiting conditions than in rich media. Although the MIC changed depending on the media for both PKZ18-22 and gentamicin, the FIC remained the same, implying the synergy between them is independent of media conditions.

The biology of the T-box mechanism has been thoroughly investigated, with extensive reviews of the genetics (reviewed in references 24 and 43) as well as biochemical and structural features (reviewed in references 26 and 50) being available. The process for tRNA recognition is a 2-step process under kinetic control that occurs as the T-box is being transcribed (30, 31, 51). The specifier loop contains a codon sequence that recognizes the cognate isoacceptor through a codon-anticodon interaction, which involves a 4° turn of the specifier loop residues. The tRNA elbow forms an additional point of contact with the apical loop of stem I (28). This conserved secondary structure and the commonality of T-box-mediated gene regulation in Gram-positive bacteria make it an appealing potential drug target, especially considering that there is an aminoglycoside binding site in the antiterminator helix (52), and the mechanism can be activated or repressed by several different antibiotics *in vitro* (53).

**FIG 5:**
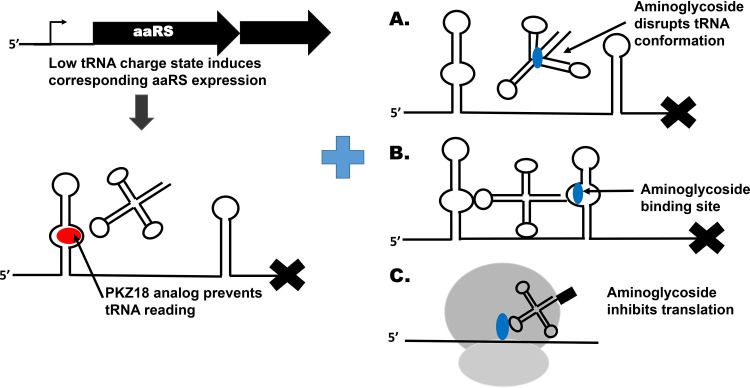
Model of synergistic interactions between PKZ18 analogs and aminoglycoside antibiotics. Low tRNA charge state induces the expression of aminoacyl tRNA synthetases that are controlled by the T-box mechanism (11 in MRSA). PKZ18 analogs cause the termination of transcription, exacerbating starvation. One or more cellular functions of aminoglycosides in combination with this can lead to synergistic interaction. (A) Aminoglycoside antibiotics can displace divalent metal cations from tRNA, which may reduce the available pool of tRNA for stabilization of T-boxes, resulting in lower levels of antitermination. (B) Aminoglycoside antibiotics such as neomycin B can bind the antiterminator helix of at least some T-boxes and may therefore affect transcriptional read-through. (C) Aminoglycoside antibiotics inhibit translation, and the combined effect of low levels of aminoacylated tRNA and reduced translational capacity leads to enhanced activity.

The mechanism of the synergy between PKZ18 analogs and aminoglycosides is not clear, but we postulate three potential explanations (Fig. 5). A partial explanation for the synergy between aminoglycosides and PKZ18 analogs may arise from the aminoglycosides’ ability to replace divalent cations on tRNA, altering the conformation of the tRNA isoacceptor (Fig. 5A) (54). Although beyond the scope of this study, it would be reasonable to expect that binding affinity, and therefore stabilization of the antiterminator by the various tRNAs, would be altered if the conformation of the tRNA is changed. Such a mechanism would involve targeting both the T-box riboswitches by PKZ18 analogs while the aminoglycosides target the tRNA ligands required to stabilize the antiterminator of the T-boxes in question. However, out of the synergistic aminoglycosides, only neomycin B is known to strongly interact with tRNA, whereas kanamycin does not. Hygromycin strongly binds tRNA but is only additive with PKZ18-22, suggesting that tRNA targeting is insufficient on its own to explain the synergy (48).

A second possibility is that aminoglycosides interact with the T-box antiterminator (Fig. 1 and Fig. 5B), as suggested by *in vitro* data (52, 53), although the binding affinity varies between T-boxes. Neomycin, however, has been shown to increase antitermination of the glycyl T-box in S. aureus *in vitro* (53), a function that is contradictory to the proposed mechanism of PKZ antibiotics. Both mass spectrometry (MS) and UV thermal stability studies indicate that PKZ18 binds stem I of the T-box and the specifier loop as targeted by computational molecular dynamics (33). Neomycin B has been found to bind the 7-nucleoside antiterminator bulge of the T-box (Fig. 1). The binding occurs in an electrostatic-dependent manner and displaces 3 to 4 monovalent cations (36). It is not known if all aminoglycosides can interact with the antiterminator, and this represents an area in need of further investigation.

A third possibility is the combined effect of depleting aminoacylated tRNAs and inhibiting translation simultaneously (Fig. 5C). There is some disagreement on the exact binding mode of aminoglycoside antibiotics on the ribosome, where two distinct binding sites include a well-characterized one on the 30S subunit and a less studied site on the 50S subunit (54–57). Neomycin and gentamicin bind the same site on the small ribosomal subunit, causing inhibition of initiation of peptide synthesis (55). However, the second binding site on the large subunit, while identical in location, differs with respect to the residues with which the antibiotics interact (56). Although the rRNA where aminoglycosides bind is highly conserved between bacterial species, there are species-specific differences in how aminoglycoside antibiotics bind the ribosome (56). Gentamicin, neomycin, and kanamycin interact with both ribosomal subunits, while streptomycin has a distinctly different binding site (44), and hygromycin has a unique chemical structure and binding mode (58), thus providing a potential explanation for the combinatorial effects with PKZ drugs for the synergistically interacting aminoglycosides that warrants further testing.

The media-dependent increase in MIC for both mupirocin and gentamicin indicates that neither targeting RNA nor depleting a sole amino acid-charged tRNA is adequate to explain the reduction in PKZ18-22 MIC in nutrient-limiting conditions. The RNA sequencing and qRT-PCR results showing that PKZ18-22 can decrease the read-through of multiple T-box controlled genes in MRSA strongly support the proposed T-box-targeting mechanism of PKZ18 drugs. Most T-boxes were affected, but the effect was especially prominent for the glycine, isoleucine, leucine, phenylalanine, serine, threonine, tyrosine, and valine T-boxes. The cysteine T-box required 8 μg/ml of PKZ18-22 to reduce read-through of the operon compared to untreated. Only 8 μg/ml of PKZ18-22 was sufficient to reduce the read-through of the alanine and histidine T-boxes compared to 4 μg/ml; however, the read-through was not reduced compared to untreated, indicating that, although almost all T-boxes were affected, the effects of PKZ18-22 vary from T-box to T-box.

This inhibition of multiple T-boxes by PKZ18 analogs has an enhanced effect. In contrast, the inhibition of the single aminoacyl-tRNA synthetase, ileRS, by mupirocin was not sufficient to enhance antibiotic activity in nutrient-limiting conditions. The RNA sequencing indicates that most of the T-boxes are targeted by PKZ18-22, while other 5′ UTRs are not affected, confirming the previously proposed mechanism that PKZ18 drugs act by suppressing T-box-regulated gene expression. The relatively high MIC values may be due to a need for ample levels of drug to the simultaneous targeting of multiple T-boxes, requiring sufficient amounts of drugs for all target T-boxes.

The only T-box-controlled gene not encoding an aaRS gene, *hom*, which codes for a homoserine dehydrogenase, was one of only two T-box-regulated genes not affected by PKZ18-22, *alaS* being the other. *Hom* expression (and its UTR expression) was very low under the conditions tested, so definitive conclusions cannot be drawn. Others have shown that daptomycin treatment causes an increase in the expression of *hom*, as well as in several other amino acid biosynthesis and transport proteins (59). Interestingly, *hom* was not among those with the most significantly changed expression in any of the treatment conditions, potentially due to differences in growth conditions. However, not all T-box-controlled genes’ expression was significantly changed, such as *hom* and *alaS*, with PKZ18-22 treatment (Fig. S2D and E in the supplemental material), and many of the T-box genes appeared as significant hits with daptomycin treatment as well (Fig. S2F) This does not, however, take gene length into account, which may, in part, explain large differences in adjusted *P* values between different T-box-controlled genes. Furthermore, not all amino acids are necessarily required at equal quantities, which, in turn, may affect the levels of aaRS expression, and compound affinity to different T-boxes may vary.

MRSA developed resistance against PKZ18-22 at a surprisingly low frequency compared to most clinically used antibiotics. The single PKZRSA1 is also more resistant to aminoglycoside antibiotics, but the resistance mechanism is not clear. Changes in influx or efflux were not the cause. The MIC screen of the PKZRSA1 supports this finding, as different classes of antibiotics targeting different mechanisms were tested, including many that act intracellularly such as mupirocin, tetracycline, and rifampin, but showed no change between WT MRSA and PKZRSA1. Most T-boxes are targeted by PKZ18-22, making a T-box mutation an unlikely cause of resistance due to the widespread use of the T-box in the control of several different essential genes and operons. Although more work is needed, PKZ18 analogs provide a promising avenue of targeting Gram-positive pathogens, as they are refractory to resistance, have multiple cellular targets, and can be used in combination with aminoglycoside antibiotics.

## MATERIALS AND METHODS

All chemicals, including novel PKZ18 analogs, were obtained from commercial sources. Novel analogs were selected based on availability and semblance to PKZ18 and PKZ18-22. All antimicrobials were resuspended in dimethyl sulfoxide (DMSO) unless otherwise specified and verified by mass spectrometry (MS).

### Strains and media.

A549 human lung epithelial cells (grown in Ham’s F12 media [Gibco] with 0.00146 g/liter l-glutamine and 10% fetal bovine serum [FBS; Gibco] added) and J774.16 murine macrophages (grown in J7 media with 74% Dulbecco’s modified Eagle medium [DMEM; Gibco]) with 0.584 g/liter l-glutamine added, 20% FBS, 5% NCTC-109 [Gibco], and 1% nonessential amino acids) were used for cytotoxicity assays. The following bacterial strains were used in the assays: Bacillus subtilis 168, Bacillus subtilis 1A5, Staphylococcus aureus 4220, Staphylococcus aureus N315 (MRSA isolate), and Escherichia coli BL21. Bacillus subtilis 1A5 and S. aureus N315 were used in qRT-PCR experiments. The abovementioned bacterial strains were used in other experiments as specified. Bacteria were grown on plain Luria-Bertani (LB) or brain heart infusion (BHI) agar except for MRSA, which was grown on BHI agar with 20 μg/ml erythromycin. Liquid cultures were grown in BHI or minimal media (Spizizen minimal medium for B. subtilis and SSM9PR [60] for S. aureus) with the desired antibiotic or solvent control.

### Primers.

All primers used in this study are listed in Table S1 in the supplemental material.

### Sample preparation of RNA and cDNA.

RNA extraction and cDNA preparation were conducted as previously reported (33). In short, guanidine thiocyanate (0.5 M final concentration)-treated and pelleted bacterial cultures were resuspended in TRIzol, lysed by beat beating with zirconia beads, and centrifuged. The solution was transferred to fresh tubes, and the RNA was extracted with chloroform. The RNA was then pelleted with 100% isopropanol and washed with ice-cold 70% ethanol, resuspended in Millipore water, DNase treated, and extracted with another isopropanol wash and ethanol wash. The resulting RNA was quantified using a NanoDrop 3300 (Thermo Fisher), and the quality was assessed by running 1 μg on a denaturing agarose gel. DNA contamination was tested for by PCR followed by agarose gel electrophoresis. cDNA was generated from 1 μg of high-quality RNA using the SuperScript III reverse transcriptase protocol for random primers (Thermo Fisher).

### Thermodynamic stability measurements with PKZ18 analogs.

Thermodynamic parameters were derived from four UV-monitored destabilizations and four renaturations of chemically synthesized wild-type and mutant truncated B. subtilis *glyQS* stem 1 specifier loops in the absence and presence of PKZ18 (10 mM Na_2_HPO_4_ and 10 mM KH_2_PO_4_; final pH 6.8). The absorbance was collected at 260 nm and was performed using a Varian Cary 3 UV-visible spectrophotometer equipped with a Peltier temperature control accessory. The temperature was increased at a rate of 1°C per minute from 5 to 85°C. Absorbance data at 260 nm were collected as a function of temperature at a rate of four data points per minute. All experiments were performed simultaneously with a control cell containing buffer only. The error calculated is the error of the mean.

### qRT-PCR.

The qRT-PCR and following analysis were performed as previously described (33). All primers are listed in Table S1. Either 2-fold or 10-fold dilutions of cDNA were used, and amplification was monitored with Evagreen dye. The expression was normalized to either 23S (B. subtilis 1A5) or 16S (MRSA) and reported as percentage read-through of the T-boxes tested for, calculated using the threshold cycle (2^−ΔΔCT^) method.

### Minimum inhibitory and bactericidal concentration determinations.

MICs were determined essentially as previously described (33). In short, cultures were grown overnight in the appropriate media, diluted to *A*_620_ of 0.1, grown for 3 hours, and again diluted to *A*_620_ of 0.1. Cultures were then diluted 10-fold, of which 5 μl were added to each well of the 96-well plate used for the experiment except the media control wells. The initial well inoculum was 7.5 × 10^5^ for S. aureus and 2.5 × 10^5^ for B. subtilis. The 96-well plate was organized as follows. Media (100 μl) were added to all wells. Stock solutions (100 μl) of 0.5 to 1 ml of 2 times the highest concentration of antibiotics to be tested were loaded to column 1, with two technical repeats per drug. The plate was serially diluted 2-fold from left to right, and media after the last dilution were discarded, so each well had a final volume of 100 μl. The following controls were included: vehicle control (DMSO, chloroform, ethanol, etc.), cells in plain media, and media control (no cells). The initial optical density (*A*_620_) of the 96-well plate was determined, the plate was grown for 16 to 24 hours, and at that time, the final optical density (*A*_620_) was read. The initial reading was subtracted from the final reading, and the technical replicates were averaged. MICs of PKZ18 and analogs were compared to clinically used antibiotics such as gentamicin.

For minimal bactericidal concentration (MBC) testing, the wells from the above 96-well plate, which showed no growth, were plated on drug-free rich media agar plates and incubated for 16 to 24 hours, at which point they were imaged and colonies were counted.

### alamarBlue cell redox activity.

alamarBlue was purchased (Thermo Scientific) and used according to the manufacturer’s directions. In short, 50,000 cells of either A549 or J774.16 were added into a 96-well plate in 200 μl and left to adhere overnight. As a control, we used a 96-well plate with the desired drugs at 2× the final concentrations in 160 μl without adding cells. The cells’ medium was removed, and 100 μl fresh medium was added, followed by 100 μl of 2× drug from the corresponding wells in the drug plate. Media were added to the drug plate to reduce the concentrations to 1×, and the cells were incubated at 37°C for 48 or 72 hours. alamarBlue was added at 10 μl per 100 μl, and the fluorescence of both plates at 590 nm was read after overnight incubation. The drug plate fluorescence was subtracted from the cells’ plate respective values, and the data were normalized to untreated cells’ reading. All plates included a vehicle control used as the 0-μg/ml value for each drug tested. All concentrations were tested in triplicate and included at least three biological replicates. A media control, untreated control, a killing control, and a vehicle control were always included.

The cytotoxicity of synergistic or additive MICs of PKZ18 analogs and a common antibiotic (co-MIC) on eukaryotic cells was tested on A549 cells as above, but the two plates were set up identically to the checkerboard assay so that the final concentrations tested corresponded to those tested against MRSA.

### Trypan blue cell viability counting.

J774.16 cells were grown in J7 medium from a fresh passage in a 1:4 dilution of 70 to 80% confluent cells. The cells were allowed to adhere in 6-well plates overnight. The medium was replaced, and drugs were added to desired concentrations in a total volume of 5 ml. A media control, untreated control, a killing control of Triton X-100, and a vehicle control were always included. After 48 hours treatment, medium was removed, and fresh media and Trypan blue were added 1:1 in a final volume of 400 μl. Cells, 100 to 200, from two areas of each well were counted and the data averaged and normalized against untreated cells. The vehicle control served as the 0.0-μg/ml value. At least three biological replicates were included for each drug at each concentration.

### Checkerboard assay and FIC.

S. aureus N315 cultures were prepared identically to MIC determination in BHI media. The co-MICs were determined using the checkerboard method (61–64). In short, antibiotics to be tested with PKZ18 analogs were diluted 2-fold top to bottom (A to G rows) in a 96-well plate, PKZ18 analogs were diluted 2-fold right to left (columns 11-2 of the 96-well plate), and column 12 was used for vector, media, and growth controls. Each drug was therefore tested by itself and with every dilution of the other drug.

The synergistic and additive interactions were determined based on determined cutoffs (62) using the equation

$$\text{FIC}=\frac{{\text{MIC}}_{\mathrm{AB}}}{{\text{MIC}}_{A}}+\frac{{\text{MIC}}_{\mathrm{AB}}}{{\text{MIC}}_{B}}$$

where MIC*_AB_* is the lowest possible concentration of both drugs combined where there is no growth, and MIC*_A_* and MIC*_B_* are the MIC values of the individual drugs. For a synergistic drug interaction, the fractional inhibitory concentration (FIC) should be below 0.5, additive or indifferent effects are considered in an FIC range of 0.5 to <4, and an FIC value above 4 is considered antagonistic (62).

### Antibiotic resistance frequency (fluctuation assay).

Fluctuation assays were performed using S. aureus N315 and B. subtilis 168 with both PKZ18-22 and PKZ18 on BHI agar plates. Gentamicin on BHI agar was used as a control. The assays were conducted essentially as done previously by us and others (33, 65). In short, MRSA and B. subtilis were grown overnight in BHI, and 3-hour day cultures at the optical density of *A*_620_ of 0.1 were started for both organisms. The optical density after 3 hours of growth was measured; cultures were then pelleted and resuspended to give 10^11^ CFU/ml by optical density. CFU of 10^11^ per OD were plated in total; 100 μl of the cells were then plated on BHI agar containing either 128 μg/ml PKZ18 or 64 μg/ml PKZ18-22. Serial dilutions of the cells were plated on plain BHI agar in order to calculate the CFU/ml, which was calculated as the number of colonies found on the plates divided by the total number of bacteria plated. CFU is a measure of live bacteria.

### Efflux activity.

Efflux activity of the PKZ18-22-resistant MRSA was compared to WT N315. The assay was performed essentially as done by others (42). Briefly, cultures were resuspended in phosphate-buffered saline (PBS) and loaded with ethidium bromide for 30 minutes. The cells were then resuspended in cold PBS and transferred onto a 96-well plate in the presence or absence of efflux inhibitors (100 μM carbonyl cyanide *m*-chlorophenylhydrazone [CCCP] or Verapamil) and 0.4% glucose, and the efflux activity was monitored by measuring the fluorescence of ethidium bromide every 60 seconds for 60 minutes. The activity was normalized to the highest fluorescence reading observed.

### Kill curves for resistance testing.

WT MRSA and resistant strains were grown overnight in BHI and diluted to an optical density of *A*_620_ of 2.0, and both strains were split into three 10-ml cultures with either 64 or 128 μg/ml of PKZ18-22 or 10 μg/ml vancomycin added. The optical density at 620 nm was taken every hour for the first 6 hours and a final time point after 24 hours. Dilutions of 0-, 3-, 6-, and 24-h samples of PKZ18-22 treated cultures were plated on BHI agar to determine total CFU of bacteria.

### RNA sequencing and bioinformatic analyses.

RNA was extracted as specified above from two biological replicates of MRSA grown in minimal media as described above. Untreated samples were given DMSO, and the remaining samples were treated with either 4 or 8 μg/ml of PKZ18-22 or 4 μg/ml of daptomycin. rRNA was removed from the total RNA using Illumina RiboZero kit as specified by the manufacturer’s instructions. The remaining RNA was then quantified and run on an SDS-agarose gel with standards to confirm the removal of rRNA. The small RNAs were then prepared for sequencing using NEBNext Ultra II directional RNA library prep kit for Illumina according to the manufacturer’s instructions. The library was quantified and sent to the New York State Department of Health (NYSDOH) sequencing core, where the library was analyzed on a tape station prior to sequencing on an Illumina platform. A preliminary data analysis was performed using Rockhopper and visualized with IGV.

Adapters were removed, and reads were quality-trimmed to a Phred score of 20 at their ends by the BBDuk routine in BBMap v37.93 (sourceforge.net/projects/bbmap/). Reads were mapped to the assembly for S. aureus N315 with BWA v0.7.5a-r405 (66), and read counts were obtained with the multiBamCov function from bedtools v2.17.0 (67). Differential expression analyses were conducted with DESeq2 (68) in R v3.5.3 (https://www.r-project.org) on raw read counts.

Heatmaps and column graphs were generated from base reads using GraphPad Prism 8. Other plots were generated using R (69) and ggplot2 (70).

### Data availability.

RNA-seq data generated in this study are available under BioProject ID PRJNA669520.

## Supplementary Material

Supplemental file 1
