# Supplementary material for: Small-Molecule Antibiotics Inhibiting tRNA-Regulated Gene Expression Is a Viable Strategy for Targeting Gram-Positive Bacteria
Source: Antimicrob Agents Chemother. 2020 Dec 16;65(1):e01247-20. doi: 10.1128/AAC.01247-20 (PMC7927825; doi:10.1128/AAC.01247-20)
Supplement: Supplemental file 1 [file AAC.01247-20-s0001.pdf]

Supplemental Figure 1. Effect of PKZ18-22 on transcriptional read-through of T-box controlled genes in WT MRSA grown in rich media. The ile T-box regulated *ileS* and tyr T-box regulated *tyrS* transcriptional read-through after PKZ18-22 treatment for two hours as measured by qRT-PCR and normalized to an untreated sample is shown.

Supplemental Figure 2. Cytotoxicity of combinatorial treatment on eukaryotic cells. A549 human lung epithelial cells' metabolic activity as measured by Alamar Blue after 48 hours of treatment with the concentrations of PKZ18 analogs that in combination with the other antibiotics showed improved activity. A high concentration of PKZ18 analogs is shown for comparison. **A.** PKZ18-22 and gentamicin. **B.** PKZ18-53 and gentamicin. **C.** PKZ18-22 and ampicillin. **D.** PKZ18-53 and ampicillin. **E.** PKZ18-22 and chloramphenicol. **F.** PKZ18-53 and chloramphenicol. **G.** PKZ18-22 and streptomycin. **H.** PKZ18-22 and neomycin.

Supplemental Figure 3. Representative graphs of influx and efflux activity of *S. aureus* N315 and the PKZ18-22 resistant mutant. EthBr = ethidium bromide, used at 8 µg/mL. EI = efflux inhibitor (verapamil or CCCP both at 100 µg/mL). Glucose is used to induce efflux. Controls are in black and experimental is in red, closed legends represent WT and open legends represent the resistant mutant. **A.** Accumulation of ethidium bromide in the presence and absence of efflux inhibitors over 60 minutes. **B.** Efflux of ethidium bromide.

Supplemental Table 1. List of primers used. The sequence and gene target as well as the species are specified.

22

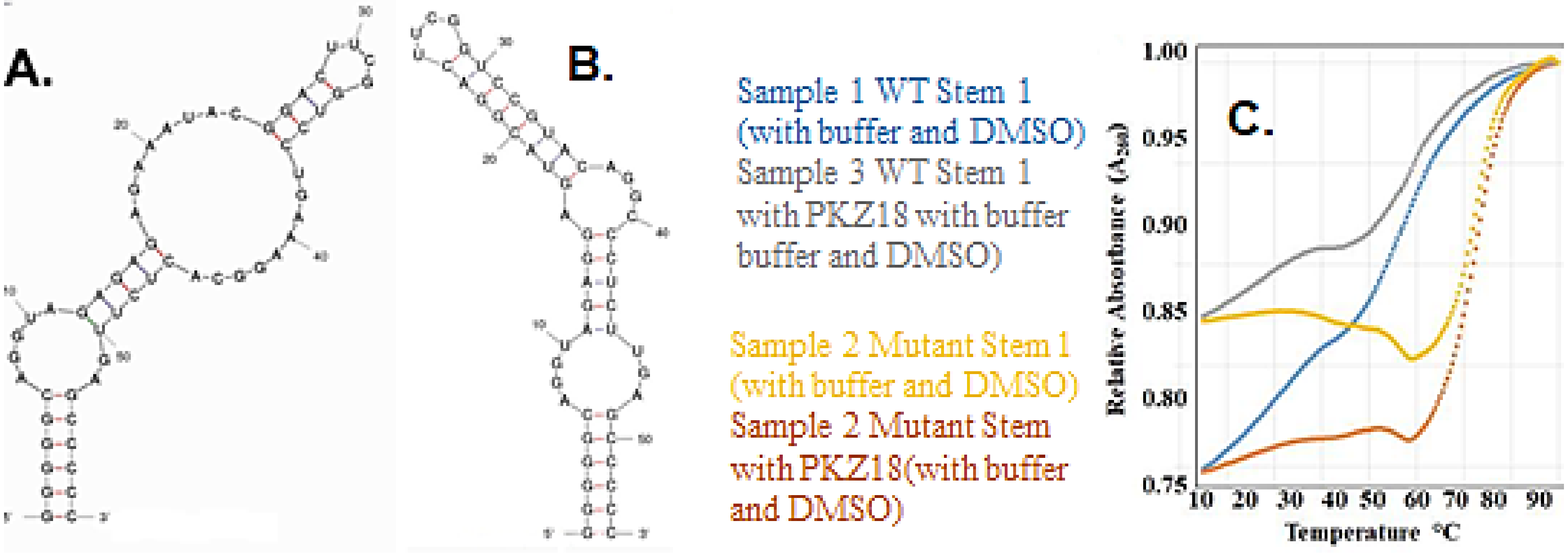

Supplemental figure 1. UV-melting assay with PKZ18. A. Native construct of gly T-box. B. Truncated construct of gly T-box missing the specifier loop. C. UV-melting curves of the two RNA constructs with and without PKZ18.

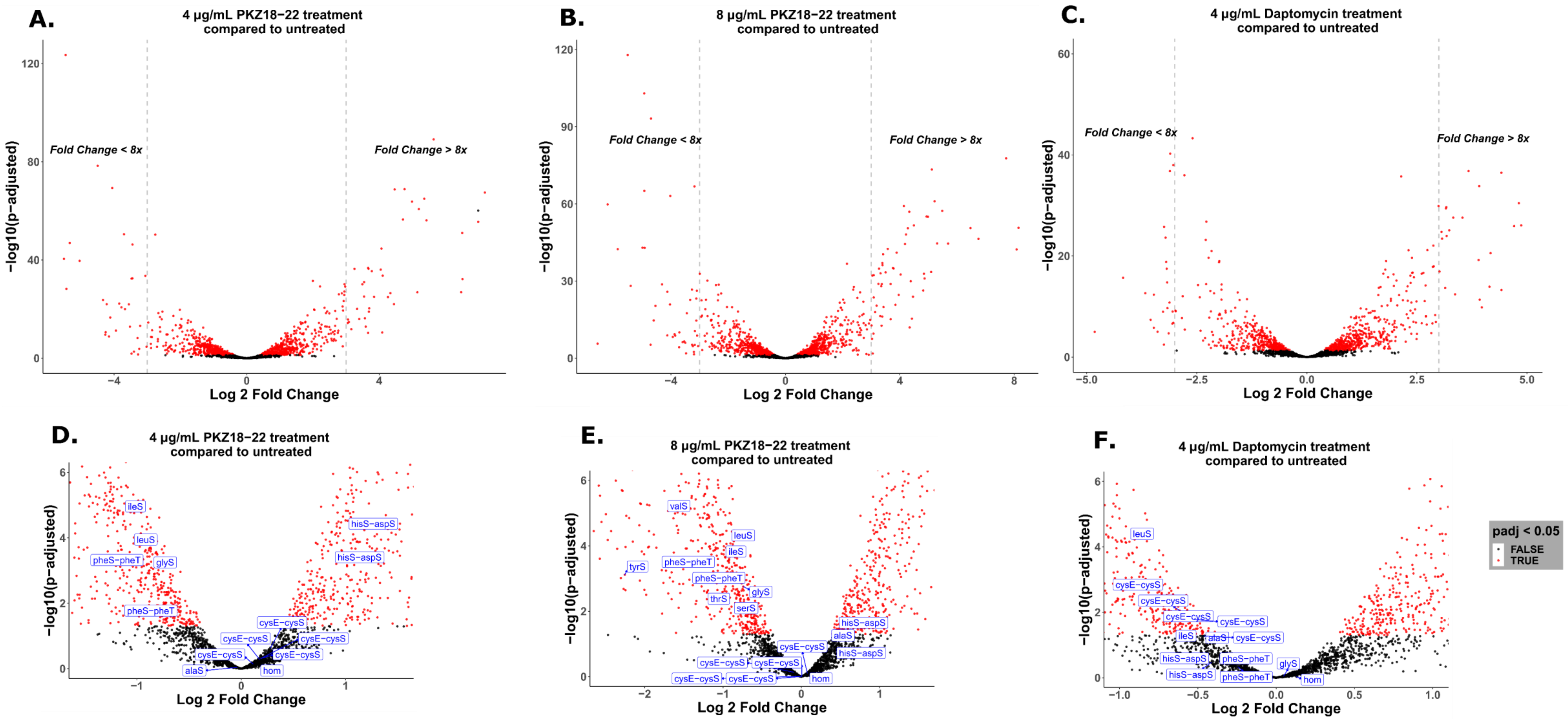

Supplemental Figure 2. Volcano plots of p-adj value plotted against fold change. T-box controlled genes are labelled in zoomed panels. Red: p-adj.<0.05, black: p-adj.>0.05. **A.** 4 µg/mL PKZ18-22 all genes affected. **B.** 8 µg/mL PKZ18-22 all genes affected. **C.** 4 µg/mL daptomycin all genes affected. **D.** Zoom in of 4 µg/mL PKZ18-22 treatment. **E.** Zoom in of 8 µg/mL PKZ18-22 treatment. **F.** Zoom in of 4 µg/mL daptomycin treatment.

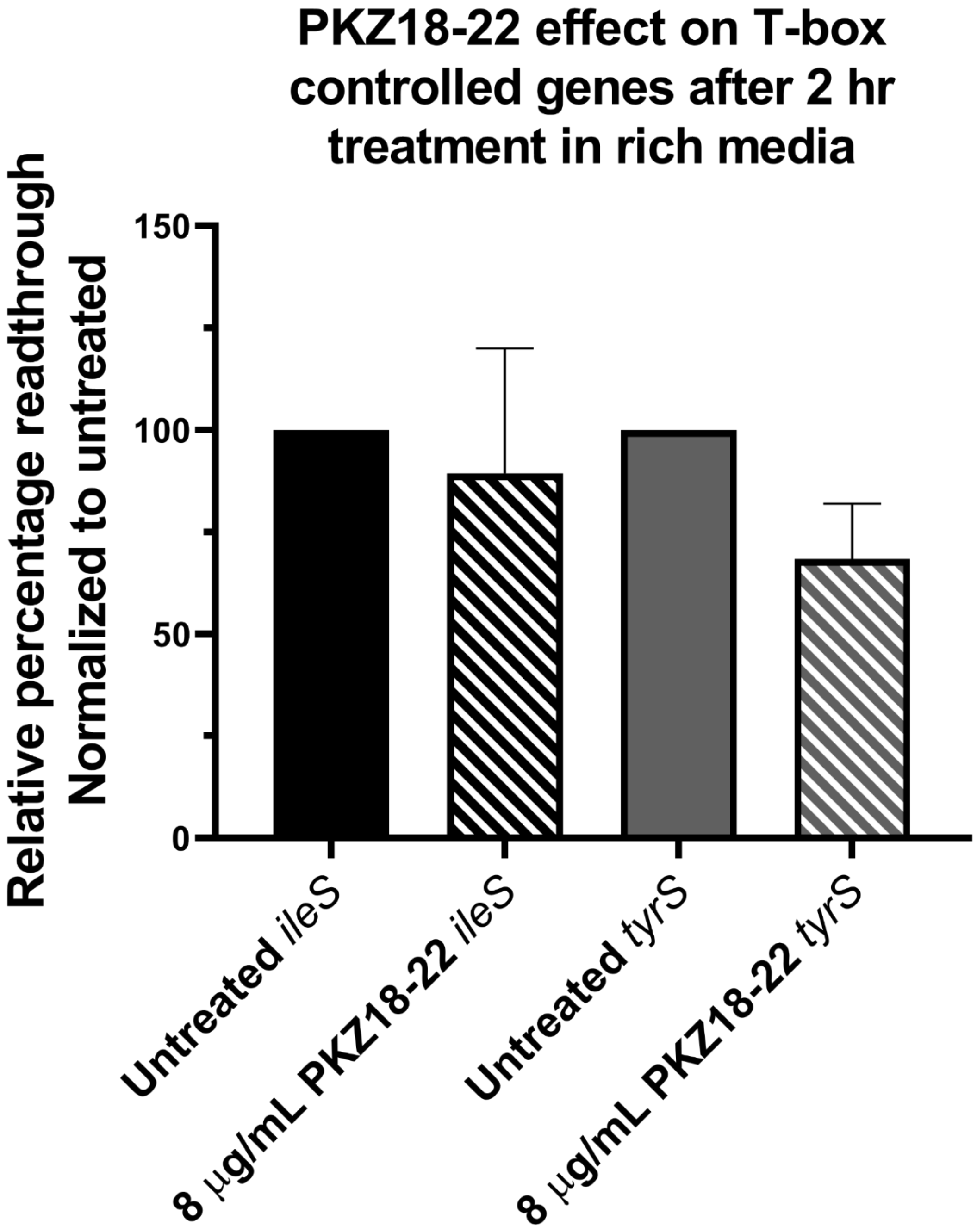

Supplemental Figure 3. Effect of PKZ18-22 on transcriptional read-through of T-box controlled genes in WT MRSA grown in rich media. The ile T-box regulated *ileS* and tyr T-box regulated *tyrS* transcriptional read-through after PKZ18-22 treatment for two hours as measured by qRT-PCR and normalized to an untreated sample is shown. (N=3)

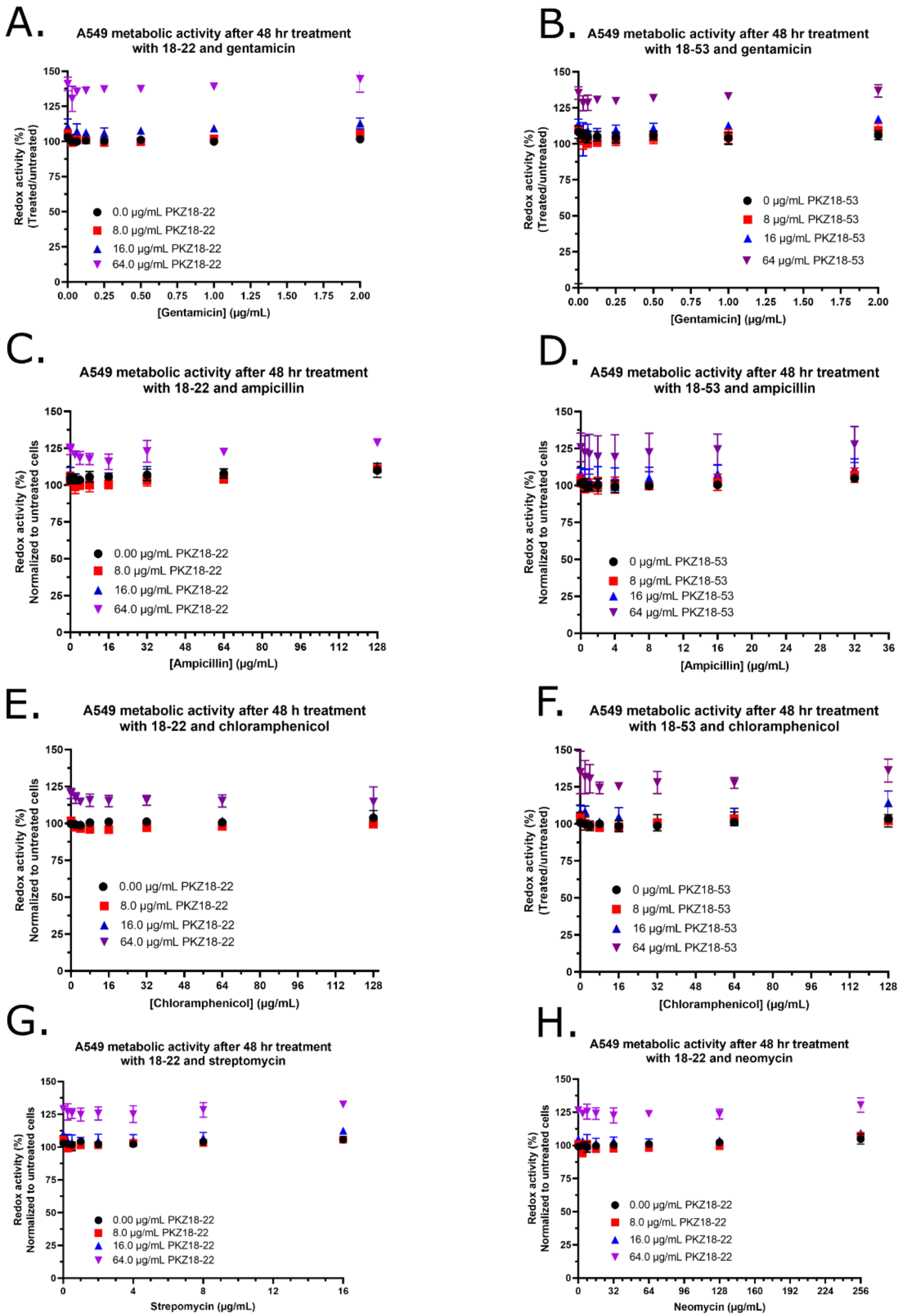

Supplemental Figure 4. Cytotoxicity of combinatorial treatment on eukaryotic cells. A549 human lung epithelial cells' metabolic activity as measured by Alamar Blue after 48 hours of treatment with the concentrations of PKZ18 analogs that in combination with the other antibiotics showed improved activity. A high concentration of PKZ18 analogs is shown for comparison. **A.** PKZ18-22 and gentamicin. **B.** PKZ18-53 and gentamicin. **C.** PKZ18-22 and ampicillin. **D.** PKZ18-53 and ampicillin. **E.** PKZ18-22 and chloramphenicol. **F.** PKZ18-53 and chloramphenicol. **G.** PKZ18-22 and streptomycin. **H.** PKZ18-22 and neomycin.

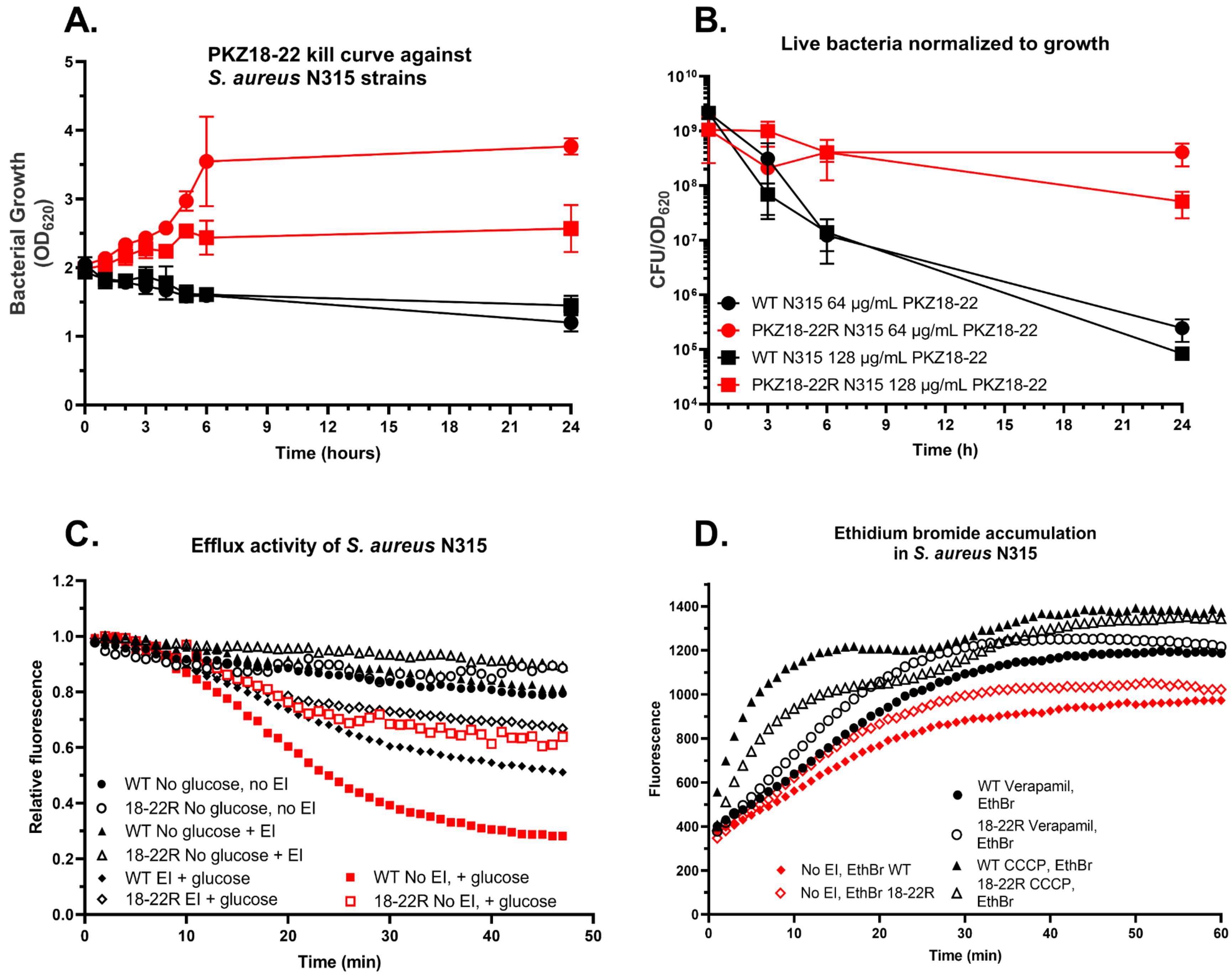

Supplemental Figure 5. Characterization of resistant mutant. **A.** Kill curve of mutant (red) and WT MRSA (black) with either 64 (circles) or 128 µg/mL (squares) PKZ18-22. (N=3 or more) **B.** Comparison of live bacteria as measured by total CFU plating normalized to growth from various timepoints from **A.** (N=2 or more) For **C.** and **D.** **r**epresentative graphs of influx and efflux activity of *S. aureus* N315 and the PKZ18-22 resistant mutant are shown where EthBr = ethidium bromide, used at 8 µg/mL. EI = efflux inhibitor (verapamil or CCCP both at 100 µg/mL). Glucose is used to induce efflux. Controls are in black and experimental is in red, closed legends represent WT and open legends represent the resistant mutant. **C.** Accumulation (influx) of ethidium bromide in the presence and absence of efflux inhibitors over 60 minutes. **D.** Efflux of ethidium bromide.

Supplemental Table 1 Primers

Name	Gene Target	Sequence 5'-3'
KM4827	B. subtilis glyQS ORF forward	ggtccagtcagggctgtgtgcttatgc
KM4828	B. subtilis glyQS ORF reverse	agccactttccacggctcaggacc
KM5298	B. subtilis glyQS UTR forward	gcggtgtaagctaaggatg
KM5299	B. subtilis glyQS UTR reverse	ttccaccctagttgctga
KM4994	B. subtilis rrnA – 23S forward	ggcttgcctcttggggttgta
KM4995	B. subtilis rrnA – 23S reverse	ggcttgcctcttggggttgtaggacactc
KM5316	B. subtilis rrnA – 16S forward	acgtgtagcggtgaaatg
KM5317	B. subtilis rrnA – 16S reverse	tggactaccagggtatctaatc
KM5679	S. aureus rrnA – 16S forward	GAGTGCAGAAGAGGAAAGTG
KM5680	S. aureus rrnA – 16S reverse	CGTCAGTTACAGACCAGAAAG
KM5683	S. aureus rrnA – 23S forward	TGAGGGTAGCGGAGAAAT
KM5684	S. aureus rrnA – 23S reverse	CAGTGCCCTACCTCCAATATC
KM5685	S. aureus rrnA – 23S forward	AGGATAGGTAGGAGCCTTTG
KM5686	S. aureus rrnA – 23S reverse	TAAGTGGTGCGGGTTAGA
KM5724	S. aureus ileS T-box forward (5'UTR)	AGGTAATGGTGAGAGCCTAGT
KM5725	S. aureus ileS T-box reverse (5'UTR)	GCGGCACCACCCTTATTAACTC
KM5728	S. aureus ileS forward	CAGCTGAATTCCGTGAGAAATG
KM5729	S. aureus ileS reverse	AAGTCACCACGAACACCTAAA
KM5836	S. aureus tyrS T-box forward (5'UTR)	cctcctaaattacgtatagtataccatatttaagc
KM5837	S. aureus tyrS T-box reverse (5'UTR)	gacctctccattttaaatcttcaattaatacattcgtc
KM5838	S. aureus tyrS T-box sequencing (5'UTR)	caattaatacattcgtcattattatttcctcc
KM5899	S. aureus tyrS T-box forward (5'UTR)	GAAATCATCAGAGAGCTAGTGGT
KM5900	S. aureus tyrS T-box reverse (5'UTR)	CGTAGGACATVTTAGTTATAAAGGTG
KM5901	S. aureus tyrS forward	TCAGAAGAACGTGTGCTACAA
KM5902	S. aureus tyrS reverse	CACCATGGTCTGTTCCAAATCC
